# A review of the current treatment methods for posthaemorrhagic hydrocephalus of infants

**DOI:** 10.1186/1743-8454-6-1

**Published:** 2009-01-30

**Authors:** David Shooman, Howard Portess, Owen Sparrow

**Affiliations:** 1 Department of Neurosurgery, Wessex Neurological Centre, Southampton General Hospital, Tremona Road, Southampton, Hampshire, SO16 6YD, UK; 2 Children's X-Ray, Department of Radiology, Southampton General Hospital, Tremona Road, Southampton, Hampshire, SO16 6YD, UK

## Abstract

Posthaemorrhagic hydrocephalus (PHH) is a major problem for premature infants, generally requiring lifelong care. It results from small blood clots inducing scarring within CSF channels impeding CSF circulation. Transforming growth factor – beta is released into CSF and cytokines stimulate deposition of extracellular matrix proteins which potentially obstruct CSF pathways. Prolonged raised pressures and free radical damage incur poor neurodevelopmental outcomes. The most common treatment involves permanent ventricular shunting with all its risks and consequences.

This is a review of the current evidence for the treatment and prevention of PHH and shunt dependency. The Cochrane Central Register of Controlled Trials (CENTRAL, The Cochrane Library) and PubMed (from 1966 to August 2008) were searched. Trials using random or quasi-random patient allocation for any intervention were considered in infants less than 12 months old with PHH. Thirteen trials were identified although speculative interventions were also evaluated.

The literature confirms that lumbar punctures, diuretic drugs and intraventricular fibrinolytic therapy can have significant adverse effects and fail to prevent shunt dependence, death or disability. There is no evidence that postnatal phenobarbital administration prevents intraventricular haemorrhage (IVH). Subcutaneous reservoirs and external drains have not been tested in randomized controlled trials, but can be useful as a temporising measure. Drainage, irrigation and fibrinolytic therapy as a way of removing blood to inhibit progressive deposition of matrix proteins, permanent hydrocephalus and shunt dependency, are invasive and experimental. Studies of ventriculo-subgaleal shunts show potential as a temporary method of CSF diversion, but have high infection rates.

At present no clinical intervention has been shown to reduce shunt surgery in these infants. A ventricular shunt is not advisable in the early phase after PHH. Evidence exists that pre-delivery corticosteroid therapy reduces mortality and IVH and there may be trends towards reduced disability in the short term. There is also evidence that postnatal indomethacin reduces IVH but with no effect on mortality or disability. Overall, there is still no definitive algorithm for the treatment of PHH or prevention of shunt dependence. New therapeutic approaches in neonatal care, including those aimed at pre-empting PHH, offer the best hope of improving neurodevelopmental outcomes.

## Introduction

Intraventricular haemorrhage (IVH) remains a serious complication of premature birth. Despite many treatment options, there is still no consensus on the management of post-haemorrhagic hydrocephalus (PHH) in the very low birth weight (VLBW) baby. Although improvements in obstetric and perinatal care have decreased the incidence and severity of IVH in low-weight preterm infants from 40–50% in the 1980s, to 20–25% in the 1990s [1,2], the problem still remains important as the survival rate of very immature newborns increases [3]. Moreover, there is a direct correlation between increasing prematurity and severity of the IVH. A 1998–2000 study reported IVH grade III in 32% of premature infants born at 24–26 weeks' gestation and IVH grade IV in 19% of this group, whereas in infants born at 31–32 weeks gestation the incidences of IVH grades III and IV were 11% and 5% respectively [4]. Additionally, the percentage of patients developing hydrocephalus secondary to this haemorrhage varies greatly. In the last 20 years different medical and surgical treatments have been put forward to prevent both the occurrence of haemorrhage and the development of hydrocephalus. In this study we reviewed the literature on the different treatments used to control and treat PHH.

## Background

With improvement in neonatal intensive care, more children with PHH are surviving. Murphy *et al*. provided evidence that posthaemorrhagic ventricular dilation in the 1990s had a more aggressive course than previously [1]. This is presumably due to the increasing survival of infants of progressively lower birth weight and gestation followed by improvements in neonatal care, such as the widespread use of antenatal steroids and surfactants. However the less well-developed brain in these very premature neonates may also be more readily damaged by haemorrhage or increased intracranial pressure (ICP).

Haemorrhage into the ventricles of the brain is amongst the most serious complications of preterm birth despite improved survival rates. Large IVH has a high risk for neurological disability, and more than 50% of these children go on to develop progressive ventricular dilation [3]. Extreme prematurity associated with PHH results in high morbidity and considerable mortality [1,5]. The hydrocephalus is usually ascribed to fibrosing arachnoiditis, meningeal fibrosis and subependymal gliosis, which impairs the flow and reabsorption of cerebrospinal fluid (CSF). Initially, multiple blood clots may obstruct the ventricular system or CSF reabsorption channels, such as from thrombus formation within the cisterna magna [6]. This leads to a chronic arachnoiditis of the basal cisterns, involving deposition of extracellular matrix proteins in the foramina of the fourth ventricle and in the subarachnoid space [7,8].

Recent experimental studies have suggested that acute parenchymal compression and ischaemic damage, with increased parenchymal and perivascular deposition of extracellular matrix proteins, are the important contributors to the development of arachnoiditis and hydrocephalus [8]. Transforming growth factor beta (TGF-beta) is a cytokine that up regulates the production by fibroblasts of extracellular matrix proteins. TGF-beta is involved in the initiation of wound healing and fibrosis [9]. TGF-beta elevates the expression of genes encoding fibronectin, various types of collagen [10,11], and other extracellular matrix components [12]. There is evidence that TGF-beta may be a mediator of the pathological process [13].

Previous studies suggest that these changes take place over weeks. Heep *et al*. in 2004 confirmed that TGF-beta CSF concentrations are in fact not elevated in the acute phase of fibroproliferative reactions in patients with PHH [14]. However, this study demonstrated that vascular endothelial growth factor (VEGF) is highly expressed in the CSF of neonates with PHH and may serve as an indicator of brain injury from progressive ventricular dilation. Further studies confirmed that intraventricular blood and ventricular expansion may have adverse effects on the immature periventricular white matter by a variety of other mechanisms, including physical distortion, raised ICP [15], free radical generation facilitated by liberated iron [16], and inflammation [17]. The haemorrhage can be isolated or it can rupture through the ependymal lining into the ventricular system. If the haemorrhage is large it may extend into the parenchymal tissue adjacent to the germinal matrix. The majority of haemorrhages occur within 72 hours of birth.

Despite all the difficulties in isolating a pathogenetic mechanism, there is little doubt that IVH is associated with damage to periventricular white matter and this damage is exacerbated by the development of hydrocephalus. Combinations of pressure, distortion, ischaemia, inflammation, and free radical-mediated injury are the main contributors. Importantly, damage to white matter accounts for the high frequency of cerebral palsy and poor neurodevelopmental outcome, in this group of infants. The aim of much of past and ongoing research is to identify mechanisms and mediators of hydrocephalus and white matter damage, which may enable new treatments that avoid permanent hydrocephalus, with its potential neurological sequelae, and avoid shunt dependence.

Treatment is more difficult than with other types of hydrocephalus. The large amount of blood and protein in the CSF, combined with the small size and instability of the patient, makes early ventriculoperitoneal (VP) shunt operations unrewarding, due to the high incidence of blockage and infection [18]. There is a considerable complication rate throughout later life from such surgery, and the child is generally permanently dependent on the shunt system [19]. Several studies act as testament to this difficulty. Pikus *et al*. [20] evaluated 52 patients with Grade IV IVH and progressive hydrocephalus treated between 1977 and 1987. These patients averaged 6.9 shunt revisions over 18 years. Mortality was 60%; 78% of survivors had intellectual function more than 2 standard deviations below the mean for age and all survivors had some form of spasticity. Boynton *et al*. [18] reported outcomes for 50 preterm infants with PHH, 92% with Grade III or Grade IV haemorrhages. Mortality was 7% with a median shunt revision rate of 4 per patient and shunt infection rate of 19% per procedure, with a failure rate of 92% for those treated in the first 3 weeks of life. Neurodevelopmental outcomes included strabismus (40%), blindness (28%) and hearing loss (24%). Seizures occurred in 38% and most required long-term anticonvulsants. Additionally, significant limitations in motor function occurred in 49%. More recently, a large cohort study by Adams-Chapman *et al*. [21] into 6161 extremely low birth weight children, confirmed that those with severe IVH that require shunt insertion are at greater risk for adverse neurodevelopmental and growth outcomes at 18 to 22 months, compared with children with and without severe IVH and with no shunt. These poor outcomes reinforce the need for more alternative treatments, though they are not necessarily caused by the shunt, perhaps being due to the underlying processes leading to shunt dependence.

### Anatomy and pathophysiology

Germinal matrix haemorrhage (GMH) and IVH are the most common and most important events that cause neurological injuries in preterm neonates. The homeostatic compensatory capacity of a premature infant is limited. Fluctuations in cerebral blood pressure and flow can rupture the primitive germinal matrix vessels or lead to infarction of the metabolically active germinal matrix. The damage can extend into the periventricular white matter, resulting in significant neurological sequelae, including cerebral palsy, mental retardation, and seizures. As has been mentioned, injury to the germinal matrix results in substantial mortality and morbidity rates.

The germinal matrix is located in the subependyma of the ventricular walls. At 8–28 weeks' gestation, the germinal matrix initially produces neurons and subsequently glial cells, which migrate to populate the cerebral cortex. Involution of the germinal matrix toward the caudothalamic groove begins late in the second trimester and is nearly complete by 32 weeks' gestation [22]. The germinal matrix is metabolically active with a rich supply of blood via thin, fragile capillary networks, supplied by branches of the anterior cerebral artery. The arterioles flow from the recurrent artery of Heubner at the level of the foramen of Monro and the terminal branches of the lateral striate arteries, which are located more superiorly. Venous blood flows through the terminal vein, which drains via the internal cerebral vein into the vein of Galen [23].

The neuropathology of GMH/IVH is characterised by bleeding into the subependymal germinal matrix, with or without subsequent rupture into the lateral ventricle. The pathogenesis of GMH is multifactorial. The influences can be divided into intravascular, vascular, and extravascular factors. The autoregulation of blood flow in response to changes in blood pressure in the germinal matrix circulation is underdeveloped in premature infants, and the thin microvasculature is susceptible to rupture. The immature mesenchymal and glial supportive tissues also influence the extent of GMH. Large fluctuations in blood flow and blood pressure can lead to injury to the germinal matrix vessels and subsequent haemorrhage [24]. Moreover, the highly metabolically active germinal matrix is particularly vulnerable to hypotension and hypoperfusion, and this can lead to focal or diffuse infarction. Consequently, haemorrhage can occur in the infarcted regions after reperfusion. Haemorrhage from any cause can be confined to the subependymal layer, can extend into the ventricles or arise from the brain parenchyma [23]. The aetiology is probably due to obstruction of venous drainage by blood clot in the germinal matrix [25]. Thus, interventions aimed at prevention or treatment of IVH might be aimed at any of the above mechanisms. Sequelae of GMH/IVH include germinal matrix destruction, periventricular haemorrhagic infarction with subsequent encephalomalacia, and PHH. The major risk factors for GMH include a young gestational age, low birth weight, acute amnionitis, and not receiving antenatal steroids for at least 48 hours. Other neonatal risk factors include the use of general anaesthesia for caesarean delivery, Apgar scores that are less than 4 in the first minute or are less than 8 by 5 minutes, respiratory distress, patent ductus arteriosus, anaemia and arterial catheterisation [26-28].

More evidence for the deleterious effects of PHH comes from studies by near-infrared spectroscopy (NIRS). NIRS permits continuous measurement of changes in the cerebral concentration of oxygenated and deoxygenated haemoglobin and oxidized cytochrome oxidase at the bedside of infants. A recent study showed that cerebral perfusion, cerebral blood volume, and oxidative metabolism are all compromised in infantile PHH to the degree whereby removal of any volume of CSF led to a significant improvement in these parameters [29]. This further demonstrates how harmful the resultant PHH can be to the cerebral haemodynamics of infants.

### Diagnosis and grading of IVH and PHH

**Figure 1:**
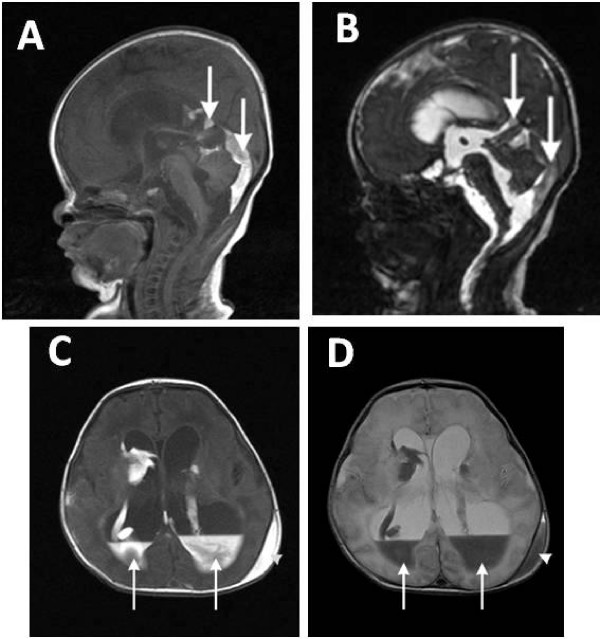
**MR images of the head of a very low birth weight infant nine days after birth**. A: Sagittal T1-weighted MRI showing layering of the intraventricular haemorrhage within the venticles and in the posterior fossa (arrows). B: CISS (Constructive Interference in the Steady State) sequence giving heavily T2-weighted high resolution images with excellent fluid contrast demonstrating the dilated ventricular system in white and the dependent haemorrhage (arrows). C: T1-weighted axial MRI scan showing layering of the intraventricular haemorrhage (arrows) and a left scalp haematoma (arrowhead). D: T2-weighted axial MRI showing layering of the intraventricular haemorrhage (arrows) and a left scalp haematoma (arrowhead). The most striking features on these scans are hydrocephalus with intracranial haemorrhage, which is seen layering within the lateral ventricles.

**Figure 2:**
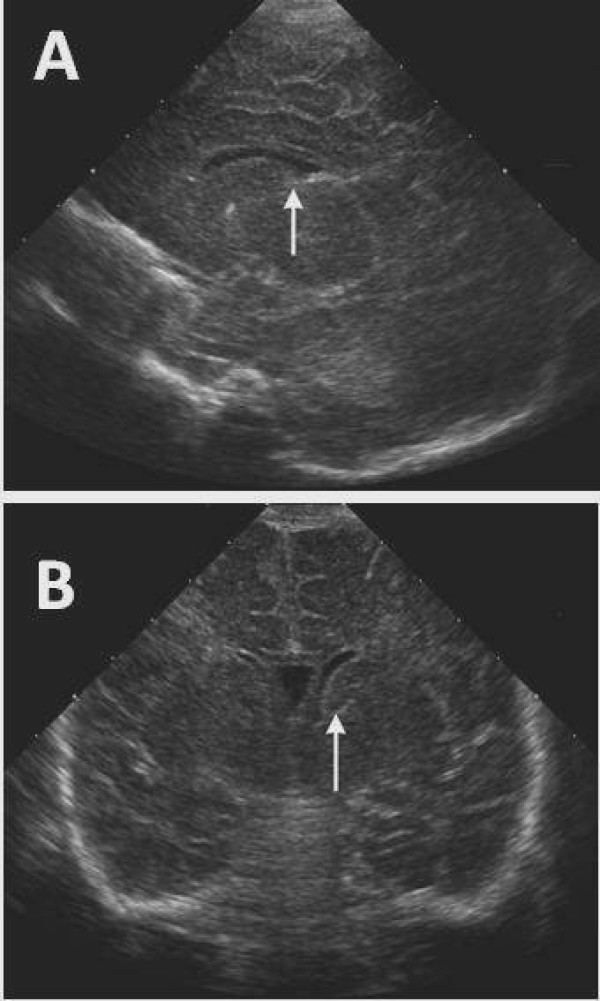
**Ultrasound images of the head of a 2 week old normal term infant scanned with an 8V5 ultrasound probe on an Acuson Sequoia 512 ultrasound machine (Siemens, Erlanger, Germany)**. A: Sagittal view, arrow indicates the caudo-thalamic groove used as a reference point. B: Coronal view, again arrow referencing the normal caudo-thalamic groove.

**Figure 3:**
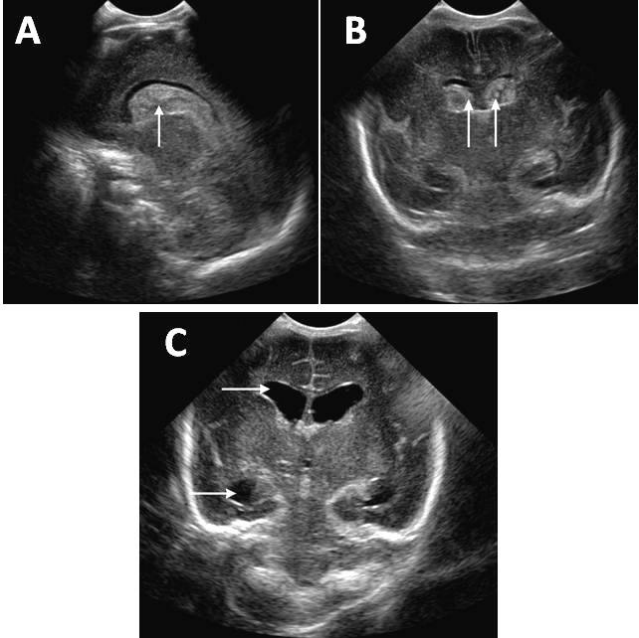
**Ultrasound images of a 2 day old premature infant born at 28 weeks scanned with a C8-5 ultrasound probe on a Philips iE33 ultrasound machine (Philips Medical Systems, Eindhoven, Netherlands)**. A: Sagittal scan presenting with bilateral grade III intraventricular haemorrhage, with blood in the caudo-thalamic groove (arrow). B: Coronal ultrasound scan presenting bilateral grade III intraventricular haemorrhage (arrows), with greater than 50% filling of the lateral ventricles. C: Coronal ultrasound scan with bilateral grade III post haemorrhagic hydrocephalus in the same infant six weeks later. The dilated anterior and temporal horns of the right lateral ventricle are apparent (arrows).

Ultrasonography (US) is the primary imaging modality for the screening and diagnosis of GMH/IVH, and computed tomography (CT) scanning and magnetic resonance imaging (MRI) are used as supplementary tools [30]. Figure 1 shows MRI sequences of a VLBW infant nine days after birth with hydrocephalus and intraventricular and caudo-thalamic groove haemorrhages. Cranial ultrasound (US) can be carried out at the cot side and does not expose the infant to ionizing radiation [31]. This enables whole populations of infants to be safely examined. The classification of IVH by Papile in 1978 was originally developed for computed tomography (CT) scanning, but has been adopted by ultrasonographers [32]. Figures 2A and 2B demonstrate coronal and sagittal US images in a normal infant, and Figures 3A–C show views of grade III IVH and subsequent hydrocephalus six weeks later.

### Four grades identified for IVH are

**Figure 4:**
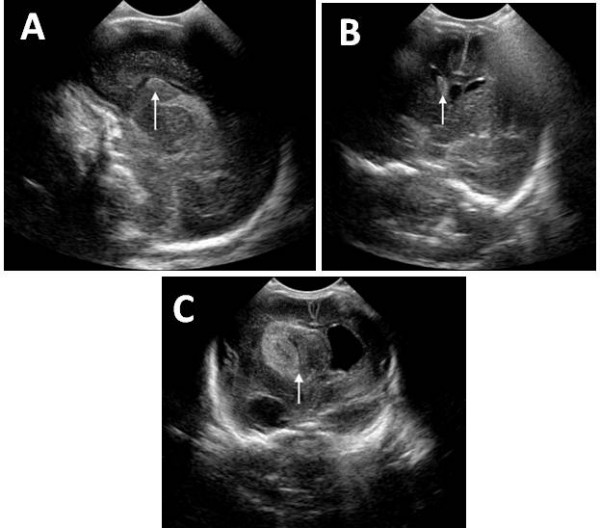
**Ultrasound images of infants with different grades of intraventricular haemorrhage scanned with a C8-5 ultrasound probe on a Philips iE33 ultrasound machine**. A: Coronal view of a 2 week old term infant with grade I intraventricular haemorrhage highlighted in the left caudo-thalamic groove (arrow). B: Coronal view of a 1 day old premature infant born at 24 weeks with grade II intraventricular haemorrhage in the right caudo-thalamic groove (arrow) which fills less than 50% of the ventricle. C: Coronal view of a 2 day old premature infant born at 24 weeks with a right grade IV intraventricular haemorrhage with hydrocephalus and extension into periventricular white matter (arrow).

Grade I or mild haemorrhage is confined to the subependymal germinal matrix with no blood clot in the ventricular lumen. Figure 4A shows a coronal US image with grade I IVH highlighted in the left caudothalamic groove.

Grade II or moderate subependymal haemorrhage involves minimal filling (10–40%) of the lateral ventricles with little or no ventricular enlargement. Figure 4B is a coronal US image pointing out grade II IVH in the right caudothalamic groove.

Grade III or severe subependymal haemorrhage is associated with substantial filling of the lateral ventricles (> 50%) with significant ventricular enlargement (Figure 3).

Grade IV or periventricular haemorrhagic infarction is IVH plus intraparenchymal venous haemorrhage. Figure 4C is a coronal US highlighting a right grade IV large IVH with hydrocephalus and extension into periventicular white matter.

Although US diagnosis of GMH is not perfect, with sensitivity of 61% and specificity 78%, the diagnosis of IVH shows high sensitivity (91%) and specificity (81%) as does identification of parenchymal haemorrhage (sensitivity 82% and specificity 97%) [33].

### Literature search

Literature searches were performed using PubMed and the Cochrane Central Register of Controlled Trials (CENTRAL, The Cochrane Library Issue 2, 2008). Randomised or quasi-randomised controlled trials were considered as primary (level 1) evidence for treatment (see additional file 1). Other small-scale studies were considered to affirm potential and understudied therapies. All types of intervention were considered in infants of less than 12 months of age with hydrocephalus following IVH.

### Shunt treatment and complications

At the present time, the best definitive treatment for hydrocephalus in preterm infants is still the VP shunt. The most suitable time for surgery is when the newborn infant exceeds 1500 g [34] and the CSF has a protein content below 200 mg/dL [35]. However, alongside poor long term outcomes, there are many problems with this method. Firstly, the surgical treatment is still complicated by high revision rates [36,37]. Secondly, the prematurity of the patients and their relatively incompetent immune system favours shunt infections. The infection rate varies across the studies and is usually between 5–15%. 3. Also increased CSF protein levels predispose to shunt obstructions which are the most common cause of shunt failure in these children [18,36-38], with the rate of shunt failure reportedly being higher in ventriculoatrial shunts [38]. Furthermore, physiologically very low ICP levels in premature infants necessitate special shunt systems, which bring more potential problems. For low birth weight infants, low pressure valves or even valveless shunts are recommended [39]. Problematically, low pressure valves may have to be converted later to higher pressure valves, or include systems with a component to prevent over drainage when upright as the child starts to sit and stand. Therefore, there is a high rate of shunt revision even in the absence of blockage, unless externally programmable valves are used. In addition, shunt over drainage with collapsed ventricles and eventual subdural fluid collections and/or secondary craniostenosis can occur in children with low pressure valve systems [37,40]. Some authors, therefore, advocate shunts with programmable valves as the first choice although there is in fact limited evidence as to the superiority of one valve over another in addressing these risks, which only underlines the uncertainties in using these devices [41,42]. There is also controversy about the setting for the surgery. Some authors advocate the neonatal intensive care unit (ICU) as the most comfortable and safest for premature patients [43]. Finer *et al. *[44] demonstrated that the unstable neonate can undergo surgery in the neonatal ICU with a surgical morbidity and mortality comparable to that seen in theatre, as transportation predisposes the critically ill neonate to hypothermia, frequently results in dislodgement of intravascular catheters, and is likely to increase postoperative pain. However, these studies apply to general procedures with no specific evidence for relevance to shunt operations. Therefore, given the high infection rate of shunting preterm neonates with PHH, this is an environment probably best limited to external ventricular drain (EVD) placement, unless evidence of safety emerges [45].

Nevertheless, VP shunting is clearly still a treatment with many problems in preterm infants with PHH, and prevention or alternative therapies are undoubtedly needed.

### Medical and minimally invasive treatments

#### Diuretic therapy

Medical treatment of PHH consists of oral or intravenous administration of drugs that reduce CSF production. Acetazolamide and furosemide, which both reduce the production of CSF, have been suggested as non-invasive therapies to reduce hydrocephalus and the need for VP shunting. There are two level 1 trials of acetazolamide and/or furosemide compared with standard therapy in infants with IVH or post-haemorrhagic ventricular dilatation (PHVD); one randomized with 16 infants [46] and the other with 177 infants [47]. Neither study showed a decreased risk for VP shunt or death with acetazolamide and furosemide therapy. In the multicentre trial with 177 infants by the International PHVD Drug Trial Group [47] acetazolamide in doses of 100 mg/kg/day, and furosemide 1 mg/kg/day were administered. None of the measures of outcome in this study suggested any advantage from drug treatment. They concluded that substantial future reductions in the adverse consequences of PHVD were most likely to come from continuing changes in neonatal care that contribute to its prevention rather than to its treatment. In total, 65% of infants receiving the drug treatment died or required shunt insertion and 46% of infants in the control group died or required shunt insertion. In the drug treatment group, 79% of infants were impaired or disabled at 1 year, whereas 53% of those in the control group were impaired or disabled at 1 year. The CNS infection levels were similar in both groups. Additionally, this large trial demonstrated that acetazolamide and furosemide treatment resulted in a borderline increase in the risk for motor impairment at one year (RR 1.27, 95% CI 1.02 – 1.58; RD 0.16, 95% CI 0.02 – 0.31). However, when the combined outcomes of death, developmental delay, disability or impairment at one year were considered there was no significant increase in risk. Another finding was that diuretic treatment increased the risk for nephrocalcinosis (RR 5.31, 95% CI 1.90 – 14.84; RD 0.19, 95% CI 0.09 – 0.29) and a meta-analysis has confirmed the significance of this result [48]. Thus the diuretic therapy increased nephrocalcinosis and biochemical anomalies, which led to the cessation of treatment. Additionally, the available evidence supports the assertion that diuretic therapy increases the risk of motor impairment and disability [48]. As the results clearly showed a worse outcome in the drug-treated infants, the data-monitoring committee prematurely halted the trial.

The rationale for treatment with these drugs is to decrease the rate of CSF formation, but they have other effects. There is the potential to cause cerebral vasodilatation and impairment of autoregulation, leading to enhanced risk of secondary cerebral injury. Moreover, data from experimental studies suggest that acetazolamide may be toxic to the developing oligodendrocyte [49]. The adverse effects seen in these trials, however, are probably best explained by alterations in cerebral perfusion pressure [47]. Therefore, available evidence shows that acetazolamide and furosemide do not reduce the need for VP shunting in infants with PHH, being neither effective nor safe.

#### Fibrinolytic agents

Treatments involving fibrinolytic agents carry a high risk of triggering new haemorrhages, but in recent years their use has been taken up again in combination with ventricular drains. The effect of intraventricular streptokinase was determined on the risk of permanent shunt dependence, neurodevelopmental disability or death in neonates at risk for, or actually developing PHH. Two randomized trials, both evaluating intraventricular streptokinase in 12 infants who developed PHVD were identified [50,51]. When intraventricular streptokinase was compared with conservative management of PHVD, the numbers of deaths and babies with shunt dependence were similar in both groups. No information on the effect of intraventricular streptokinase on disability is available. There is cause for concern about meningitis and secondary IVH, but numbers are insufficient to quantify the risks [50,51]. Overall, therefore, intraventricular fibrinolytic therapy with streptokinase starting before one month of age in infants developing PHVD is not recommended.

#### Drainage, irrigation, and fibrinolytic therapy (DRIFT)

Recently, Whitelaw and colleagues [52] piloted a method involving intraventricular administration of tissue plasminogen activator (tPA) and 72 hours drainage via two ventricular catheters (one frontal on the right, and one occipital on the left). The procedure, DRIFT (drainage, irrigation, and fibrinolytic therapy) attempted to remove intraventricular blood and the cytokines that are associated with hydrocephalus before it could become established. They randomly assigned 70 preterm infants who had gestational ages of 24 to 34 weeks with progressively enlarging cerebral ventricles after IVH to two groups, (1) drainage, irrigation, and fibrinolytic therapy to wash out blood and cytokines and (2) tapping of CSF by reservoir as required, to control excessive expansion and signs of raised ICP (standard treatment). The results were that DRIFT did not reduce VP shunt surgery or death in preterm infants with ventricular dilation after IVH when compared with tapping of CSF to control excessive head expansion or raised ICP. Tapping a ventricular reservoir was relatively safe and effective in controlling hydrocephalus even in extremely small infants. Unfortunately, it was concluded that secondary IVH is a factor that counteracts any possible therapeutic effect from washing out old blood. Therefore, the conclusion was that this innovative intervention could not be recommended until more objective evaluations can provide less equivocal findings.

Therefore, neither the DRIFT procedure nor acetazolamide and furosemide are effective in reducing the need for shunting, and both forms of therapy have adverse effects. It may be that the results to date of intraventricular fibrinolytic therapy have been negative because the treatment (starting nearly two weeks after birth) had been administered too late. Fibrosis, deposition of extracellular matrix proteins and chronic inflammatory changes may already have become irreversible. Furthermore, very low levels of plasminogen and the presence of Plasminogen Activator Inhibitor-1 (PAI-1) [53] would be expected to limit the fibrinolytic effect of intraventricular streptokinase. As we have alluded to, there is evidence that the cytokine, TGF-beta plays a major role in the development of PHH. Whitelaw *et al*. [13], further demonstrated that intraventricular injection of tPA, on its own, increases the concentration of TGF-beta in ventricular CSF. This could help to explain the failure of intraventricular injection of fibrinolytic agents to prevent hydrocephalus. Thus, therapeutic strategies need to continue to consider ways of removing, blocking or preventing release of this and other cytokines such as VEGF.

### Preventative measures for IVH

As has been mentioned, approximately 80% of IVH occurs by 72 h after birth but a considerable proportion of IVH is visible on the first scan within a few hours [54]. This means that interventions to prevent IVH should ideally be commenced prior to, or immediately after delivery.

#### Postnatal phenobarbital

The rationale for administration of postnatal phenobarbital to prevent IVH in low birth weight infants is underpinned by: a) the observation that phenobarbital may dampen fluctuations in systemic blood pressure in premature infants [55], b) evidence that treatment with phenobarbital reduces the incidence of intracranial haemorrhage in newborn beagles made hypertensive with phenylephrine [56], c) experimental evidence that barbiturates can partially protect the brain against hypoxic-ischaemic damage [57] and d) the suggestion that phenobarbital's free radical scavenging capacity may protect after hypoxia-ischaemia [58]. However, the results of three trials which used posthaemorrhagic ventricular dilatation or hydrocephalus as an outcome, reported no significant difference between the control or treatment groups [59-61]. The typical estimates from a meta-analysis of these studies [25] provide little evidence of a reduction in the risk of posthaemorrhagic ventricular dilatation (typical relative risk 0.89, 95% CI 0.38, 2.08, typical risk difference -0.01, 95% CI – 0.08, 0.06). Moreover, a large double-blind randomised controlled trial by Kuban *et al*. [62], found that in VLBW infants, phenobarbital was actually associated with an increased risk of developing any subependymal-intraventricular-intraparenchymal haemorrhage. Although they did not use PHH as an independent outcome measure, this study complements our assessment that the published evidence is very much against postnatal phenobarbital preventing IVH. Furthermore, phenobarbital suppresses spontaneous breathing in infants, causing a need for mechanical ventilation. In spite of the lack of efficacy for this intervention, there is still scope for researchers to consider perinatal intervention with other modalities.

### Surgical treatment other than shunt placement

As regards surgical treatment of PHH other than shunt surgery, some authors have recommended ventricular drains in preference to subgaleal reservoirs, due to the levels of reported infection rates, but the numbers reported are too small for any confidence [63]. The standard treatment varies from centre to centre, and few have built up a large series for analysis. The standard arms of the Ventriculomegaly Trial [64] and the PHVD Drug Trial into acetazolamide and furosemide [48] both used selective tapping of CSF to control signs of raised ICP or excessive head enlargement. Inserting a subcutaneous ventricular access device (VAD), to facilitate repeated tapping of adequate CSF volumes is widely practiced, without having been tested by a randomised trial. All these measures are considered here.

#### Lumbar puncture and ventricular taps

Repeated early lumbar puncture (LP) or ventricular taps have been advocated as a way of avoiding hydrocephalus and protecting the brain from excess pressure. It was thought that the risk of hydrocephalus and the need for a VP shunt might be reduced by the removal of protein and old blood from the CSF. In practice, the decision to employ this strategy is taken from head circumference changes, fontanelle examination, neurological signs, and sequelae of raised ICP and from US measurement of ventricular dilatation. In line with the Levene criteria for ventricular indices [65], intervention usually occurs when the ventricle reaches a diameter > 4 mm above the 97th percentile [66], although this is controversial as dilatation may already be too great for subsequent taps to be effective and there have been reports of better outcomes with taps performed before this cutoff [67]. A common practice is to remove sufficient fluid to render the fontanelle soft and depressed, typically in the region of 10 ml/kg/tap [68]. This hypothesis has been tested in four randomised trials involving premature infants in whom IVH (with or without established enlargement) was diagnosed by US. In total, four controlled trials, were identified, three being randomised and the fourth using alternating allocation [64,69-71]. Two trials evaluated repeated LPs in neonates with IVH [70,71] and two trials evaluated repeated CSF tapping in infants with IVH followed by progressive ventricular dilatation [64,69]. The total number of infants in the four studies was only 282, with 157 coming from the Ventriculomegaly Trial. None of the trials found a significant effect from CSF tapping on: a) need for shunt, b) death, c) major disabilities in survivors, d) multiple disabilities in survivors, or e) death or disability. Similarly, a meta-analysis by Whitelaw of the results of all included trials showed no significant effect of CSF tapping on any of these outcomes [72]. Nevertheless, this does not take into account the potential benefit of tapping to delay shunt implantation until such time as the skin in VLBW babies matures sufficiently to reduce the risk of shunt erosion.

Repeated CSF tapping of preterm infants carries a theoretical risk of introducing infection. None of the infants in Dykes' 1989 study [69] developed CSF infection during tapping but 11 of the 157 infants in the ventriculomegaly trial developed CSF infections, all having had some CSF taps (the infants in the control group were eventually tapped if they developed symptoms or signs of raised ICP) [64]. CSF infection (meningitis/ventriculitis) can be a serious adverse effect of early repeated CSF tapping. There is no information about the frequency of needle-track lesions from repeated ventricular taps, though anecdotal evidence does exist. For example, a large (presumed traumatic) aneurysm of the pericallosal artery in an infant has been observed after repeated ventricular taps by the senior author (OS) (unpublished). Although it was a reasonable hypothesis that early CSF tapping would reduce ICP and remove protein and blood to clear the CSF pathways, the meta-analysis by Whitelaw of the four controlled trials failed to demonstrate any evidence of benefit [72]. Moreover, the secondary risk of infection and the discomfort of the procedures were reiterated.

Assessment of the infants in the ventriculomegaly trial at twelve months included an analysis in two groups: a) infants who had a cerebral parenchymal lesion visible on US at entry, and b) infants with no cerebral parenchymal lesion at entry. In the group with parenchymal brain lesions at entry, there was a difference in neurodevelopmental outcome at 12 months in favour of those who had early CSF tapping [64]. This difference just achieved significance at the 5% level, but caution was expressed in the paper as to whether this finding could be due to chance. The neurodevelopmental examination at 30 months in the infants with parenchymal brain lesions at entry, showed no difference between the two treatment groups. Thus, this paper emphasised the importance of basing clinical recommendations on consistent findings among large groups of subjects.

Therefore, there is no evidence that early tapping of CSF by LP or ventricular tap reduces the risk of poor neurodevelopmental outcome, shunt dependence or death after 30 months follow up. The use of repeated taps was associated with an increased risk of central nervous system infection. Thus, the use of early tapping is not a recommended treatment modality, and removing CSF should be reserved for cases where there is symptomatic raised ICP, for example while temporising before more definitive CSF diversion.

#### External ventricular drainage

After LPs prove to be unsuccessful, external ventricular drainage (EVD) is often the next invasive step in the management of PHH. The catheter is typically inserted into the dilated anterior horn of the right lateral ventricle under sterile conditions. The end of the proximal catheter is subcutaneously tunneled to a site on the scalp (or body) distant from the initial incision and connected to an adjustable drainage system. EVD appears to be much more effective than repeated LPs or ventricular taps in evacuating sufficient volumes of CSF.

The infection rate with EVD ranges from unacceptable levels [36] to very low rates reported by some authors, despite long periods with an EVD [38,63]. One study by Cornips *et al*. [63] reported no infections in 14 patients with an EVD for PHH. They put this down to vigilant monitoring and insertion of the EVD within the relatively clean and stable environment of a neonatal ICU. Studies by Berger *et al*. [73] and Rhodes *et al*. [74] each with 37 patients, reported infection rates of 5.4% and 6%, respectively. Reinprecht *et al*. [38] studied 42 preterm patients recording a 7.1% infection rate. Weninger *et al*. [75] cultured CSF and/or the tip of the ventriculostomy catheter in each of their 27 patients, reporting a 26% contamination rate but with no clinical or laboratory evidence of ventriculitis. Importantly, these studies confirmed infection via CSF drainage catheters as an independent predictor for poor neurodevelopmental outcomes [3].

Other problems such as over drainage and the development of subdural hygromas can occur but may be minimised by careful control of ICP. EVD also has the advantage of allowing easy intrathecal administration of drugs. The effect of EVD on shunt dependency and neurological outcome is at present not actually known. Long standing EVD may encourage the need for subsequent shunt insertion by decreasing natural CSF reabsorption. On the other hand, the need for permanent shunting may be reduced by the continuous removal of bloody and protein rich CSF. Nevertheless, the rate of permanent shunting after EVD is 64–68% [63,74].

#### Subgaleal shunts

Ventriculo-subgaleal shunts have been recommended as a more physiological and less invasive means of achieving CSF diversion until VLBW infants gain adequate weight, and the blood and protein levels in CSF are low enough, before a permanent shunt can be placed. In one study of this procedure to evaluate the effectiveness and complications over a 1-year period [76], the ventriculo-subgaleal shunt controlled the progression of hydrocephalus in all 6 premature infants, as assessed by clinical and imaging parameters, and a permanent shunt was avoided in 1 patient (16.6%). However, 4 patients developed shunt infections, 1 involved the ventriculo-subgaleal shunt itself, and 3 occurred immediately after conversion to VP shunt. The total infection rate in these cases was two thirds, though there was only a 1% shunt infection rate for primary implantation in their institution at that time. Therefore, according to this study, placement of ventriculo-subgaleal shunts for interim CSF diversion in neonates with posthaemorrhagic hydrocephalus is effective as a temporary method of CSF diversion. However, it is associated with a worryingly high CSF infection rate. This study suffers from a small sample size in an understudied area, and thus cannot yet be treated as a standard therapeutic option, though it seems worthy of further study.

A potential cause for infection in this study may have been CSF stasis just beneath the extremely thin skin of the premature infants, promoting colonisation by skin flora, as all the infections were staphylococcal. It is postulated that CSF sampling before conversion to a permanent shunt and replacement of the proximal hardware, which may have been *in situ *for a prolonged period, may decrease the infection rates. However, until this is researched further, it cannot be a recommended treatment.

#### Subcutaneous reservoir

The subcutaneous reservoir or VAD is another frequently used option in the management of PHH. This avoids repeated needle tracks through the brain and potential injury. Reservoirs are tapped up to three times a day and the amount of CSF removed can be adjusted to the opening pressure. An important drawback of VADs (and LPs) is that the removal of CSF is intermittent. The fluid buildup and resulting rise of ICP between taps might be detrimental. The major complications of VADs are ventriculitis, shunt infection, skin necrosis, CSF fistula, or subdural hygroma.

One recent study found hyponatraemia to be a consequence of serial CSF punctures in preterm infants with a Rickham VAD [77]. This has also been found in studies performed less recently [78], and has also been reported as a consequence of EVDs [63]. However, hyponatraemia as a consequence of CSF tapping or drainage is totally predictable and preventable, and can easily be treated with diligent monitoring and replacement [77,78]. The rates of infection in VADs reported in the literature are actually very low. However, among patients who received a subcutaneous reservoir, subsequent shunting was necessary in 75–88% [79-81].

On balance, therefore, the current evidence for VAD use is conflicting. A study by Hudgins *et al*. [80] in 149 infants with PHH recorded an 8% infection rate and concluded that the device can be recommended for several months with acceptable rates of infection, blockage and wound complications. On the other hand, a study by Richard *et al*. [82] into 64 infants recorded a 22% infection rate and did not suggest an Ommaya reservoir was beneficial in terms of mortality, prevention of shunt placement or neurological outcome. Nevertheless, a contemporary study by Brouwer *et al*. [83] assessed two separate groups, 26 infants admitted during 1992–7 and 50 admitted during 1998–2003, treated with a ventricular reservoir. Their results suggest a learning curve reflecting the benefits of experience, whereby significantly fewer complications were recorded in the second period. It is also important to emphasise that the repeated punctures need to be undertaken with meticulous aseptic technique in order to minimise introduction of organisms from the skin. They suggest that in experienced hands, a ventricular reservoir is a safe treatment to ensure adequate removal of CSF in preterm infants with PHH.

#### Ommaya reservoir followed by shunt or ETV

A 2007 study has taken this a step further and evaluated treating PHH with an Ommaya reservoir, followed by VP shunt and/or endoscopic third ventriculostomy (ETV) [84]. There were 18 premature babies affected by IVH grades II to IV implanted with a reservoir. CSF was intermittently aspirated percutaneously from the VAD according to clinical requirements and ultrasonographic follow-up. Fourteen of the patients suffered progressive ventricular dilatation and underwent VP shunt implantation (5 patients) or ETV (9 infants). One of the infants died during the study, and at the end of the follow-up period, 10 out of 17 premature neonates affected by PHH were shunt free (59%). They concluded that the combination of Ommaya reservoir, VP shunt, and the aggressive use of ETV, as either a primary treatment or as an alternative to shunt revision, allowed for a significant reduction of shunt dependency in a traditionally shunt-dependent population. Therefore, this is a promising approach, deserving of further study.

### Improvements in antenatal and perinatal care

A general decrease in IVH has been noted in developed countries over the last 10 years despite an increase in survival of very immature infants. This may in fact be due to dedicated neonatal teams who are becoming experienced at handling premature babies.

Maternal corticosteroid administration before preterm delivery has been mainly responsible for this decrease in IVH as demonstrated in a 2006 Cochrane review [85]. Indeed, in a meta-analysis of previously published trials, antenatal corticosteroids have been confirmed to reduce the incidence of IVH in premature children. The combined odds ratio for the development of IVH was 0.38 when comparing antenatal corticosteroids with placebo [86]. A promising randomised control trial by Liu *et al*. [87] evaluated whether combined antenatal corticosteroid and vitamin K administration has a benefit, over and above that of corticosteroid or vitamin K used alone, in reducing the frequency and the degree of periventricular-intraventricular haemorrhage (PIVH) in premature newborns of less than 35 weeks' gestation. More infants in the control group had grades III or IV intracranial haemorrhage after birth (p = 0.049). After antenatal supplement of dexamethasone and vitamin K1, both the total incidence of PIVH and the frequency of severe PIVH decreased significantly. Thus, many of the advances for treating PHH may be pre-emptive and the focus for research into improving antenatal care and interdisciplinary approaches is encouraging.

Of the other pharmacological interventions assessed, antenatal indomethacin appeared promising. However, results of a multicentre trial by Pringle *et al*. [88] of indomethacin in 1200 infants with birth weights < 1100 g showed that the reduction in IVH was not accompanied by an improvement in survival without disability, and a similar trial from Dani *et al*. [89] showed ibuprofen to be ineffective in preventing IVH. Nevertheless, although the long-term benefits are unproven, the therapeutic value of indomethacin in reducing the incidence of IVH, and hence PHH, should be noted [90]. It has been demonstrated in multiple single-centre studies to decrease the incidence of IVH in VLBW preterm infants [91,92] and, therefore, presumably, improves long term outcome. Furthermore, initial studies of neuromuscular paralysis with ventilation seemed to have a favourable effect on incidence and severity of IVH, but there is still uncertainty regarding its long term pulmonary and neurological effects [93]. Evidently, stronger evidence is required before this can be recommended.

Future surgical trends for PHH might include contributions from the fields of robotics, image-guided neuroendoscopy, '*in utero*' therapies and the applications of stem cell research, though these remain speculative [94].

### Implications for practice

On the basis of available evidence, routine use of early CSF tapping for infants at risk of, or actually developing PHH cannot be recommended. It would seem wise to be conservative in the management of infants developing PHH to reduce the risk of iatrogenic damage. Routine scanning is important to determine the presence or absence of parenchymal brain lesions, as they affect prognosis. The infant should be followed with repeated measurements of head circumference and ventricular width, as well as clinical examination of neurological status and fontanelle tension. Despite the lack of evidence from randomised trials, CSF drainage is obviously important if there is symptomatic raised ICP, as shown by either a deterioration in neurological signs with a tense fontanelle [72], or directly measured CSF pressure > 12 mm Hg [72]. Some evidence supports CSF drainage also in the presence of either decreasing diastolic velocities on cerebral artery Doppler waveforms [68] or deteriorating sensory evoked potentials [72]. However, neither of the latter can be regarded as routine clinical tools, so require further evaluation before contemplating adding to the routine criteria for draining CSF.

Many infants need few, if any CSF taps but continue to expand their ventricles and heads at a rate that is clearly above normal. If this excessive expansion continues until the clots have largely absorbed, shunt surgery should be considered. The surgeon may wish to postpone surgery if the infant is still extremely small, particularly if skin quality is poor, if there is infection, or if the CSF still has visible blood or high protein. If it has been necessary to tap the CSF repeatedly, then the case for earlier shunt surgery is stronger, because repeated CSF taps, particularly ventricular taps, create morbidity.

## Conclusion

Neither treatment by repeated lumbar or ventricular tapping, nor the use of acetazolamide and furosemide to reduce CSF production, are proven to prevent the need for shunt surgery or improve neurodevelopmental outcomes. Additionally, both have appreciable adverse effects. Phase 1 clinical trials of intraventricular fibrinolytic therapy and a small randomised trial have not given encouraging results. There is insufficient evidence for postnatal phenobarbital in preventing IVH, and drainage, irrigation, and fibrinolytic therapy to wash out blood and cytokines is both highly invasive, experimental and showed no benefit, so cannot be recommended routinely. However, it may be that earlier use of an implanted reservoir for tapping could improve outcomes. Overall, therefore, although the proportion of premature infants suffering IVH has declined, PHH is still a serious complication for which we do not have a definitive treatment. Research clearly needs to focus more on the mechanisms of hydrocephalus and radically new therapeutic approaches will have to be considered. In the interim, advances in paediatric intensive care and neonatal medicine offer hope in preventing PHH.

## Abbreviations

CSF: cerebrospinal fluid; DRIFT: drainage, irrigation and fibrinolytic therapy; EVD: external ventricular drain; GMH: germinal matrix haemorrhage; ICU: intensive care unit; IVH: intraventricular haemorrhage; LP: lumbar puncture; PHVD: post-haemorrhagic ventricular dilatation; PHH: post-haemorrhagic hydrocephalus; PIVH: periventricular-intraventricular haemorrhage; TGF-beta: transforming growth factor beta; tPA: tissue plasminogen activator; US: ultrasound; VAD: ventricular access device; VEGF: vascular endothelial growth factor; VP: ventriculoperitoneal.

## Competing interests

The authors declare that they have no competing interests.

## Authors' contributions

DS conceived of the review, carried out the literature search. DS and OS wrote the paper in tandem. HP compiled the images. All authors have read and approved the final version of the manuscript.

## Supplementary Material

Additional file 1 **Table 1**. Review of the literature. Click here for file
