# Supplementary material for: A review of the current treatment methods for posthaemorrhagic hydrocephalus of infants
Source: Cerebrospinal Fluid Res. 2009 Jan 30;6:1. doi: 10.1186/1743-8454-6-1 (PMC2642759; doi:10.1186/1743-8454-6-1)
Supplement: Additional file 1 — Table 1. Review of the literature. [file 1743-8454-6-1-S1.doc]

**Table 1. Review of literature**

- Only articles with level 1 evidence

| **Author** | **Intervention** | **Method** | **Participants** | **Outcomes** |
| --- | --- | --- | --- | --- |
| **Libenson**  **1999 [1]** | Acetozolamide or Furosemide | Open randomised controlled trial | N of randomised patients = 16 (10 in treatment group, 6 in control group)  Infants with birthweights < 1500 g with IVH on ultrasound followed by i) enlargement of ventricular width to 5 mm over 2 SDs and ii) raised ICP defined as either an increase in ICP of 20 mm water or an absolute  ICP exceeding 90 mm water.  ICP was measured either by the Ladd fiberoptic non-invasive method or by  lumbar puncture. | Ventriculoperitoneal shunt.  Nephrocalcinosis.  Death |
| **PHVD (Posthaemorr-hagic ventricular dilatation) Trial Group**  **1998/2001 [2]** | Acetozolamide or Furosemide | Open randomised controlled trial | N of randomised patients = 177 (88 in treatment group, 89 in control group)  Infants aged < 3 months with IVH on ultrasound followed by progressive dilatation until ventricular width was > 4 mm over the 97th centile | Death  Ventriculoperitoneal shunt  Disability at 1 year  Vineland social maturity scale. Motor disability was defined as motor score < 70.  Global delay was defined  as an overall Vineland score < 70.  Sensorineural deafness requiring a hearing aid.  CNS infection  Nephrocalcinosis  Duration of hospitalisation after trial entry.  Supplemental oxygen |
| **Anwar 1985 [3]** | Daily lumbar puncture starting at 7 - 10 days. CSF was drained until flow stopped. Lumbar punctures were continued until the ventricular size decreased, remained unchanged for 2 consecutive weeks or if the infant developed hydrocephalus requiring a ventricular drain or shunt. | Open randomised clinical trial | 47  Preterm infants with grade 3 or 4 intraventricular haemorrhage on ultrasound scan | Hydrocephalus was defined as a progressive increase in ventricular size as measured by ultrasound, in association with either signs of increased ICP or an increase in head circumference >2 cm/week for at least 2 weeks.  Death before discharge from hospital.  Death. |
| **Dykes**  **1989 [4]** | Daily lumbar punctures, taking enough CSF to lower the CSF pressure by half. Volumes ranged from 2 - 21 ml. Duration 1 - 3 weeks. | Open randomised clinical trial | 53  Neonates with asymptomatic severe posthaemorrhagic hydrocephalus | Hydrocephalus management failure was defined as increasing head circumference, progressive decrease in cortical mantle (eg occipital cortical mantle < 1 cm), signs of raised ICP.   Death during follow-up.  Assessment at 3 - 6 years into no major handicap, single system disability and multiple disability. |
| **Mantovani**  **1980 [5]** | Daily lumbar punctures starting 24 hours after diagnosis of IVH. 3 - 5 ml of CSF was removed daily. Lumbar punctures were continued until the CSF was clear and protein concentration was < 180 mg/dl. | Open clinical trial with alternation of treatment | 48  Infants weighing less than 2000g with grade 2 or 3 intraventricular haemorrhage on CT scan | Hydrocephalus was defined as 2 CT scans with progressively enlarging ventricles.   Death before discharge from hospital. |
| **Ventriculo- megaly**  **1994 [6]** | Repeated lumbar puncture taking as much CSF as possible, maximum 2 % body weight carried out daily or less frequently to prevent further increases in ventricular size. If not more than 2 ml of CSF could be obtained, ventricular tapping was carried out in the same way and often enough to hold the ventricular width constant. | Open randomised multicentre clinical trial at 15 neonatal intensive care units in England, Ireland and Switzerland. Randomization by telephoning and registering the infant before hearing the allocation. | 157  Neonates with intraventricular haemorrhage, with progressive increase in ventricular size and whose ventricular width had increased to 4 mm over the 97^th^ centile. | Permament shunting was carried out if there was failure to control head size despite medical management or if repeated tapping was necessary for more than 4 weeks.  Death during follow up.  Neurodevelopmental assessment was carried out at 12 months post term.  Neurodevelopmental status examined at 30 months by a developmental pediatrician. Death during follow-up. |
| **Ruth 1988 [7]** | Phenobarbitone.  2 loading doses of phenobarbital 15 mg/kg i.v. were given 4 hours apart. Maintenance treatment with phenobarbitone 5 mg/kg per day was started 24 hours after the first dose and continued for 5 days. | Open randomised controlled trial | Infants with birthweights below 1501g and gestational age 25 weeks or more, less than 4 hours old. Infants  with malformations or maternal barbiturate treatment were excluded. N = 101. 111 infants were originally  enrolled but 10 were excluded (7 in the phenobarbitone group and 3 in the control group) either because  the gestational age was < 25 weeks or because of congenital anomaly | Cerebral ultrasound scans were carried out on days 1,3,5 and 7 and then weekly. Intraventricular hemorrhage  was graded according to the Papile scale. Neurodevelopmental assessment at 27 months of age. Neonatal  death, postnatal death, mechanical ventilation (total and > 7days), pneumothorax. |
| **Donn 1981 [8]** | Phenobarbitone  Two loading doses of 10 mg/kg phenobarbital each administered intravenously 12 hours apart.. Maintenance  doses of 2.5 mg/hr every 12 hours were begun 12 hours after. Doses were adjusted to maintain serum  concentrations in the 20-30 micrograms/ml range for 7 days | Randomised controlled trial | Infants with birthweights below 1500g, admitted to the NICU within 6 hours, without congenital malformations  and where the mother had not received barbiturates during pregnancy. N = 60. No information on  infants excluded or lost after enrolment. | Outcomes Papile grade of intraventricular hemorrhage on ultrasound, ventriculomegaly, mechanical ventilation, pneumothorax  requiring drainage, hypercapnia (pCO2 > 60 mm Hg), hypotension ( systolic blood pressure 10  mm Hg below expected value or impaired perfusion), bicarbonate therapy, death. |
| **Anwar 1986 [9]** | Phenobarbitone  Two loading doses of phenobarbital 10 mg/kg intravenously starting before 6 hours of age and the second  loading dose 12 hours later, followed by a maintenance dose of 2.5 mg/kg every 12 hours for 7 days.  Maintenance doses were adjusted to achieve trough phenobarbitone concentrations of 20 - 30 mg/l | Open randomised controlled trial | Preterm infants with a birthweight below 1500g with no congenital malformations and no maternal phenobarbitone  administration. N = 58 | Papile grade of intraventricular hemorrhage by ultrasound on days 1,3,7, posthaemorrhagic hydrocephalus,  death. It is not clear that the ultrasonographers were blind to treatment allocation. |
| **Luciano 1997 [10]** | Intraventricular streptokinase after intraventricular hemorrhage  Insertion of a percutaneous ventricular catheter via the anterior fontanelle. Streptokinase 20,000 units/day  was given intraventricularly for 96 hours. CSF drainage was performed several times a day to prevent raised  intracranial pressure. | Open randomised controlled trial | 12 newborn infants who were shown by ultrasound to have intraventricular hemorrhage followed by pro-  gressive enlargement to 4 mm over the 97th centile for ventricular width | Death, insertion of a ventriculoperitoneal shunt, meningitis and secondary intraventricular bleeding. |
| **Yapicioglu 2003 [11]** | Lumber puncture to remove 5-10ml of CSF, followed by insertion of a percutaneous ventricular catheter via  the anterior fontanelle, and removal of a further 5ml of CSF. Streptokinase 25,000 units over 3 days at 0.5ml/h  was given intraventricularly. CSF drainage of 5-10ml of CSF was performed once a day. Intraventicular  vancomycin (1mg/day) for infection prophylaxis. | Open randomized trial | 12 newborn infants who developed posthaemorrhagic hydrocephalus. | Third ventricular size, death, insertion of a ventriculoperitoneal shunt, meningitis and secondary intraventricular bleeding. |
| **Whitelaw**  **2007 [12]** | DRIFT (Drainage, irrigation, and fibrinolytic therapy) | Randomised control trial | 70 preterm infants who had gestational ages of 24 to 34 weeks and were progressively enlarging their cerebral ventricles after intraventricular haemorrhage to either (1) drainage, irrigation, and fibrinolytic therapy to wash out blood and cytokines or (2) tapping of cerebrospinal fluid by reservoir as required to control excessive expansion and signs of pressure (standard treatment). | Death, insertion of a ventriculoperitoneal shunt, at 6 months |
| **Liu 2006 [13]** | Combined antenatal corticosteroid and vitamin K administration over and above that of corticosteroid or vitamin K used alone, in reducing the frequency and the degree of periventricular-intraventricular hemorrhage (PIVH) in premature newborns less than 35 weeks' gestation | Randomised control trial | 280 pregnant women were randomly allocated into five groups according to the in-patient sequence. | Incidence, frequency, severity of PIVH |

1. Libenson MH, Kaye EM, Rosman NP, Gilmore HE: **Acetazolamide and furosemide for posthemorrhagic hydrocephalus of the newborn**. *Pediatr Neurol* 1999, **20**:185-191.

2. **International randomised controlled trial of acetazolamide and furosemide in posthaemorrhagic ventricular dilatation in infancy. International PHVD Drug Trial Group**. *Lancet* 1998, **352**:433-440.

3. Anwar M, Kadam S, Hiatt IM, Hegyi T: **Serial lumbar punctures in prevention of post-hemorrhagic hydrocephalus in preterm infants**. *J Pediatr* 1985, **107**:446-450.

4. Dykes FD, Dunbar B, Lazarra A, Ahmann PA: **Posthemorrhagic hydrocephalus in high-risk preterm infants: natural history, management, and long-term outcome**. *J Pediatr* 1989, **114**:611-618.

5. Mantovani JF, Pasternak JF, Mathew OP, Allan WC, Mills MT, Casper J, Volpe JJ: **Failure of daily lumbar punctures to prevent the development of hydrocephalus following intraventricular hemorrhage**. *J Pediatr* 1980, **97**:278-281.

6. **Randomised trial of early tapping in neonatal posthaemorrhagic ventricular dilatation: results at 30 months. Ventriculomegaly Trial Group**. *Arch Dis Child Fetal Neonatal Ed* 1994, **70**:F129-136.

7. Ruth V, Virkola K, Paetau R, Raivio KO: **Early high-dose phenobarbital treatment for prevention of hypoxic-ischemic brain damage in very low birth weight infants**. *J Pediatr* 1988, **112**:81-86.

8. Donn SM, Roloff DW, Goldstein GW: **Prevention of intraventricular haemorrhage in preterm infants by phenobarbitone. A controlled trial**. *Lancet* 1981, **2**:215-217.

9. Anwar M, Kadam S, Hiatt IM, Hegyi T: **Phenobarbitone prophylaxis of intraventricular haemorrhage**. *Arch Dis Child* 1986, **61**:196-197.

10. Luciano R, Velardi F, Romagnoli C, Papacci P, De Stefano V, Tortorolo G: **Failure of fibrinolytic endoventricular treatment to prevent neonatal post-haemorrhagic hydrocephalus. A case-control trial**. *Childs Nerv Syst* 1997, **13**:73-76.

11. Yapicioglu H, Narli N, Satar M, Soyupak S, Altunbasak S: **Intraventricular streptokinase for the treatment of posthaemorrhagic hydrocephalus of preterm**. *J Clin Neurosci* 2003, **10**:297-299.

12. Whitelaw A, Evans D, Carter M, Thoresen M, Wroblewska J, Mandera M, Swietlinski J, Simpson J, Hajivassiliou C, Hunt LP *et al*: **Randomized clinical trial of prevention of hydrocephalus after intraventricular hemorrhage in preterm infants: brain-washing versus tapping fluid**. *Pediatrics* 2007, **119**:e1071-1078.

13. Liu J, Wang Q, Zhao JH, Chen YH, Qin GL: **The combined antenatal corticosteroids and vitamin K therapy for preventing periventricular-intraventricular hemorrhage in premature newborns less than 35 weeks gestation**. *J Trop Pediatr* 2006, **52**:355-359.
